# Humanizing Health and Social Care Support for People With Intellectual and Developmental Disabilities: Protocol for a Scoping Review

**DOI:** 10.2196/31720

**Published:** 2022-05-04

**Authors:** Madison Milne-Ives, Rohit Shankar, Dan Goodley, Kirsten Lamb, Richard Laugharne, Tracey Harding, Edward Meinert

**Affiliations:** 1 Centre for Health Technology University of Plymouth Plymouth United Kingdom; 2 Peninsula Medical School Faculty of Health University of Plymouth Plymouth United Kingdom; 3 School of Education University of Sheffield Sheffield United Kingdom; 4 Faculty of Health University of Plymouth Plymouth United Kingdom; 5 Cornwall Partnership National Health Service Foundation Trust Bodmin United Kingdom; 6 School of Nursing and Midwifery Faculty of Health University of Plymouth Plymouth United Kingdom; 7 Harvard TH Chan School of Public Health Harvard University Boston, MA United States; 8 Department of Primary Care and Public Health School of Public Health Imperial College London London United Kingdom

**Keywords:** developmental disabilities, intellectual disability, delivery of health care, patient care management, social work, social support, patient-centered care, empathy, respect, social care

## Abstract

**Background:**

Health care is shifting toward a more person-centered model; however, people with intellectual and developmental disabilities can still experience difficulties in accessing equitable health care. Given these difficulties, it is important to consider how humanizing principles, such as empathy and respect, can be best incorporated into health and social care practices for people with intellectual and developmental disabilities to ensure that they are receiving equitable treatment and support.

**Objective:**

The purpose of our scoping review is to provide an overview of the current research landscape and knowledge gaps regarding the development and implementation of interventions based on humanizing principles that aim to improve health and social care practices for people with intellectual and developmental disabilities.

**Methods:**

The PRISMA-ScR (Preferred Reporting Items for Systematic Reviews and Meta-Analyses extension for Scoping Reviews) and PICOS (Population, Intervention, Comparator, Outcome, and Study) frameworks will be used to structure the review. A total of 6 databases (PubMed, MEDLINE, Embase, CINAHL, PsycINFO, and Web of Science) will be searched for English articles published in the previous 10 years that describe or evaluate health and social care practice interventions underpinned by the humanizing principles of empathy, compassion, dignity, and respect. Two reviewers will screen and select references based on the eligibility criteria and extract the data into a predetermined form. A descriptive analysis will be conducted to summarize the results and provide an overview of interventions in the following three main care areas: health care, social care, and informal social support.

**Results:**

The results will be included in the scoping review, which is expected to begin in October 2022 and be completed and submitted for publication by January 2023.

**Conclusions:**

Our scoping review will summarize the state of the field of interventions that are using humanizing principles to improve health and social care for adults with intellectual and developmental disabilities.

**International Registered Report Identifier (IRRID):**

PRR1-10.2196/31720

## Introduction

### Background

Although recent efforts are being made to address health inequities, people with intellectual and developmental disabilities (IDDs) can experience difficulties in accessing high-quality care [1,2]. People with IDDs are more likely to experience earlier health limitations and have social determinants that are associated with poor health. Health care professionals (for this paper, defined as any trained individual providing some type of health or social care support to people with IDDs; eg, clinicians, health care support workers, allied health professionals, social care workers, etc) are not necessarily trained and equipped to address the needs of people with IDDs in an equitable and empowering way [1,3]. The delivery of health care has recently shifted toward a more person-centered, humanizing model [4]. Person-centered or patient-centered models of care empower patients to share responsibility for their health, enhance the personalization of care, and enable patients to make informed choices about how to manage their health needs [5]. This shift demonstrates a recognition of the importance of underpinning care practices with humanizing principles, such as empathy and respect for people’s dignity, agency, uniqueness, sense of place, personal journey, and holistic well-being [6,7].

Delivering this model of care to a high quality presents a potential challenge in people with IDDs, as they can experience barriers to equitable health care access, such as difficulty with communication and a lack of engagement. Feelings of fear or can also be exacerbated by a lack of health education, a lack of training for health care professionals, the negative attitudes of health care professionals, short consultation times, and multimorbidities (which can also increase the complexity of the care needs of people with IDDs) [8-11]. Clinically, people with IDDs have a significantly shorter life expectancy than that of the general population [12]. This is influenced by potentially preventable causes that are impacted by inequalities in the access to and provision of care, which are disproportionate to those in the general population [13]. Given the health inequities faced by people with IDDs, there is a clear need for improved means of ensuring that people with IDDs are treated with empathy, dignity, and other humanizing principles during their interactions with health and social care services.

### Rationale

Although several reviews have been conducted that focus on access to and experiences with health care among people with IDDs [14-17], none of these reviews included an overview of interventions that provide humanizing care for people with IDDs. Searching PROSPERO with the string intellectual disabilit* OR developmental disabilit* AND healthcare OR health care OR social care AND humanising OR person centred OR patient centred OR empathy OR dignity OR respect did not identify any reviews on this topic. Therefore, there is a need to conduct a comprehensive summary of what interventions are being developed and delivered to improve care for people with IDDs in accordance with humanizing principles.

Previous reviews have examined the experiences of, challenges to, and barriers for people with IDDs in accessing health care services [14-18]. The key barriers identified included difficulties with communication between patients and health care professionals; poor-quality services and a lack of services, which are often due to deficits in health care professionals’ understanding, training, and skills; and other organizational barriers related to procedures or facilities [14-18]. Although the databases and years searched were comprehensive, the reviews focused on particular services (mental health services [17], physical health care services [18], or services in acute care settings [14,15]) or populations (people with autism [18]). One review was published in 2005 and therefore does not reflect any recent changes in health care delivery [16]. Although the reviews examined the experiences of people with IDDs, they did not provide an overview of any efforts that were being made to address and mitigate the barriers identified, and they did not specifically address humanizing principles. A review by Busch et al [6] in 2019 focused on the humanization of care; it highlighted the importance of empathy and respect in patient-provider interactions and the availability of sufficient time and resources for supporting this, but it was not specific to people with IDDs. None of the reviews identified focused on informal social support for adults with IDDs. The search of PROSPERO only identified 2 planned or ongoing reviews that were relevant (a review of the accessibility of public health services for people with IDDs and a review about improving social care outcomes). However, neither review focused on humanizing principles or provided a broad overview of interventions, and one was removed for no longer being within the scope of PROSPERO.

Given the barriers and health inequities that people with IDDs experience when accessing health and social care services, an overview of the potential solutions being explored, developed, and implemented is needed. A scoping review will provide a summary of the state of the field, the inclusion of humanizing principles in interventions for people with IDDs, and the strengths and weaknesses of these interventions. This will help to inform directions for future research and development and provide an initial assessment of the potential of these interventions.

### Aim and Research Question

The aim of our review is to identify and provide an overview of interventions that promote health and social care practices for people with IDDs that are based on humanizing principles. To do this, the scoping review will focus on the following research question: *What professional interventions are being developed and delivered to promote empathy, dignity, kindness, and recognition in health and social care encounters involving people with IDDs?*

## Methods

### Overview of the Study Design

The review and search strategy were structured using the PRISMA-ScR (Preferred Reporting Items for Systematic Reviews and Meta-Analyses extension for Scoping Reviews; Multimedia Appendix 1) [19] and PICOS (Population, Intervention, Comparator, Outcome, and Study) frameworks (Textbox 1).

**PICOS (Population, Intervention, Comparator, Outcome, and Study) framework.**

**Population**

- Adults (aged >18 years) with intellectual and developmental disabilities

**Intervention**

- Formal and informal health and social care interventions and practices underpinned by the humanizing principles of empathy, compassion, dignity, kindness, and recognition (eg, referrals, assessments, clinical judgments, treatments, service management and commissioning, multiagency team working, clinical and social training, informal communities, and peer support systems)

**Comparator**

- How defined interventional types compare to the general adult population (outside of the review population’s scope); however, no comparator is required for inclusion

**Outcome**

- The primary outcome will be the inclusion of humanizing principles in professional interventions for health and social care encounters involving people with intellectual and developmental disabilities
- Secondary outcomes will include the types and characteristics of the interventions, study types, perspectives of people with intellectual and developmental disabilities about the interventions, and strengths and limitations of the interventions

**Study types**

- All study types that describe or evaluate a relevant intervention will be eligible for inclusion
- Reviews, meta-analyses, and conference abstracts or posters in which no full text is available will be excluded

### Search Strategy

Our review will search the following six databases to identify potentially relevant references: PubMed, MEDLINE, Embase, CINAHL, PsycINFO, and Web of Science. Relevant Medical Subject Headings (MeSH) terms and keywords were identified for the search based on a preliminary examination of the literature and previous reviews conducted on related topics. These terms were grouped into 3 themes and were searched by using the following search string structure: *IDD (MeSH OR keywords) AND health and social care services (MeSH OR keywords) AND humanising principles (MeSH OR keywords)* (Table 1).

**Table 1:** Search strings.

Category	Medical Subject Headings	Keywords (in titles or abstracts)
Intellectual and developmental disabilities	*Developmental Disabilities OR Intellectual Disability OR Learning Disabilities OR Autistic Disorder*	“Developmental disabilit*” OR “learning disabilit*” OR “intellectual disabilit*” OR “learning disorder*” OR “developmental disorder*” OR “special need*” OR “mental retardation” OR “mental inadequac*” OR “mental handicap” OR autis* OR “Down syndrome” OR “Down’s syndrome” OR “fetal alcohol” OR “learning difficult*” OR “congenital cognitive impairment” OR “mental impairment*” OR “pervasive development” OR “ADHD” OR neurodivers* OR “neurodevelopmental disorder*”
Health and social care services	*Delivery of Health Care OR Community Health Services OR Social Work OR Social Support OR Patient Care Management OR Patient Care Team OR Quality of Health Care OR Caregivers*	Healthcare OR “health care” OR “health and social care” OR “primary care” OR “secondary care” OR “specialist care” OR “palliative care” OR “end of life care” OR “care service*” OR “healthcare service*” OR “health care commissioning” OR “health commissioning” OR referral* OR assessment* OR diagnos* OR “clinical judgement*” OR “clinical judgment*” OR formulation OR investigation* OR treatment* OR “service management” OR “multi-agency team” OR “multi-disciplinary team*” OR “clinical training” OR “social training” OR “professional development” OR “social care” OR “social work” OR “social service*” OR “care support” OR caregiver* OR “social care commissioning” OR “social support” OR “peer support” OR “informal care” OR “information social care” OR “unpaid care” OR carer* OR “informal carer*” OR “informal social support” OR “community care” OR “care networks”
Humanizing principles	*Patient-Centered Care OR Empathy OR Respect*	Empathy OR compassion OR dignity OR kindness OR recognition OR respect OR humanis* OR humaniz* OR humanity OR “patient-centred care” OR “patient-centered care” OR “person-focused care” OR holistic OR relationship OR equity OR equality OR fair

### Inclusion Criteria

The review will include studies that describe or evaluate any type of health and social care intervention for people with IDDs (eg, training, digital support, and organizational or physical changes) that are based on humanizing principles, including empathy, dignity, and respect. Studies will be included if they describe the development of such an intervention or evaluate the implementation of such an intervention (at any stage). Therefore, all study types that describe or evaluate an intervention will be eligible for inclusion. Studies concerning people with any type of IDD will be eligible for inclusion.

### Exclusion Criteria

As the aim is to provide a broad overview of interventions and humanizing health care practices for people with IDDs, there are few exclusion criteria. The focus of the review will be on adults, so studies concerning humanizing health care interventions for children and adolescents with IDDs (aged under 18 years) will be excluded. Likewise, studies that explore humanizing health care interventions for the general population, with no specific reference to people with IDDS, will also be excluded. Studies that do not describe the development or evaluation of a specific humanizing intervention (or a set of interventions) for adults with IDDs will be excluded (eg, reviews, meta-analyses, and conference abstracts or posters with no full-text versions). To ensure that the review examines recent interventions and practices, the search will be limited to the previous 10 years; articles published before 2011 will be excluded. Studies published in languages other than English will be excluded, as the review team has limited ability to effectively undertake the analysis of such studies.

### Screening and Article Selection

References will be stored and any duplicates will be automatically removed using the citation management software EndNote X9 (Clarivate). The first screening will be performed by inputting keywords related to the inclusion and exclusion criteria into EndNote X9’s search function. Two independent reviewers will screen the remaining titles and abstracts and then the full texts. The reasons for exclusion at the full-text screening stage will be recorded. Any disagreements on eligibility at either stage will be discussed by the two reviewers, and a third reviewer will be consulted if agreement cannot be reached. A PRISMA (Preferred Reporting Items for Systematic Reviews and Meta-Analyses) flow diagram will be used to record the details of the screening and selection process (and reasons for exclusion) to ensure reproducibility and transparency.

### Data Extraction

The two reviewers will extract predetermined outcomes from the full texts (Textbox 2). As in the article selection process, any disagreements between the reviewers will be discussed, and they will be resolved by a third reviewer if necessary.

**Article information and data to be extracted.**

**General study information**

- Year of publication
- Sample size (if applicable)
- Study type
- Target population (eg, people with a specific developmental disability)

**Health and social care practices and interventions**

- Category of intervention (health care, formal social care, or informal social care)
- Type of intervention
- Aim of intervention
- Humanizing principle(s) that the practice or intervention is based on
- Brief description of intervention (features and components)

**Evaluation of intervention**

- Strengths of the intervention
- Limitations of and barriers to the intervention
- Perspectives of people with intellectual and developmental disabilities (if reported)

### Data Analysis and Synthesis

A large variety of study types, measures, and outcomes is expected. As such, a descriptive analysis will be used to provide an overview of the different types of health and social care interventions via a 3-pronged approach. The interventions will be categorized based on their main area of focus as follows: health care, formal social care, or informal social care. On the basis of these categories, a thematic analysis of the evaluations of the interventions will be conducted to summarize the common strengths and weaknesses of, and perceptions toward, the interventions. Common themes will also be explored across the three categories to determine similarities, differences, and gaps in the inclusion of humanizing principles in interventions across different care contexts. Any qualitative data related to the perspectives of people with IDDs that are included in the studies reviewed will also be summarized by using a thematic analysis.

## Results

The full scoping review has not yet begun. It will be started in October 2022, and it is expected to be completed and submitted for publication by January 2023.

## Discussion

### Comparison to Prior Work

Recent reviews related to the access of health care services by people with IDDs identified a variety of barriers [15,17,18], but no reviews were identified that examined efforts to address these barriers by using humanizing principles. Our scoping review will add to the field by providing a summary of the current state of the field of research regarding the interventions that aid the humanizing of health and social care for adults with IDDs.

### Limitations

One limitation of the scoping review is that a risk of bias assessment will not be performed on the studies. Risk of bias assessments are not a standard requirement for scoping reviews [19]; however, the lack of a risk of bias assessment limits the ability to examine research gaps related to research quality, which could provide further insight on areas for improvement in the design, development, and evaluation of humanizing interventions for health and social care for people with IDDs. Another limitation is that the scoping review methodology does not include searching for grey literature. Grey literature will be excluded to keep the broad scope of the review manageable and to focus on evaluations that have been peer reviewed, since an independent quality assessment will not be conducted. However, this means that the review has the potential to overlook some promising interventions that have been developed but not formally described or evaluated.

### Conclusions

By providing a clear overview of what is currently being explored, the strengths and weaknesses of interventions, and the gaps in the field, our scoping review will help to inform the design and development of interventions and health and social care practices that are based on humanizing principles to ensure that people with IDDs are treated with dignity, empathy, and respect. The health inequities that people with IDDs face, their higher likelihood of needing care, and the shift toward more person-centered health care make this issue particularly important to address. A clear understanding of what efforts are being made in this area will help to identify good practices and areas for improvement that will enable future interventions to facilitate more humanizing care and treatment. Using established and developing networks and publishing reviews to broadcast current findings and enhance our understanding of the current state of the field are just 2 of the many possible avenues for influencing health practitioners’ practices. Once current strategies and interventions have been identified and examined, future work will have a solid base upon which to design improved interventions and implement the learnings from the review into clinical and social care practices. Conclusions that reflect the data, acknowledge the limitations of the scoping review, and indicate key areas for future research will be drawn and disseminated via journal publication. The findings will also be summarized in plain English for their distribution to any relevant clinical or governmental stakeholders that are identified during the review.

## Appendix

Multimedia Appendix 1 PRISMA-ScR (Preferred Reporting Items for Systematic Reviews and Meta-Analyses extension for Scoping Reviews) checklist.

## Abbreviations

IDD: intellectual and developmental disability
MeSH: Medical Subject Headings
PICOS: Population, Intervention, Comparator, Outcome, and Study
PRISMA: Preferred Reporting Items for Systematic Reviews and Meta-Analyses
PRISMA-ScR: Preferred Reporting Items for Systematic Reviews and Meta-Analyses extension for Scoping Reviews
